# Clinical utility of measuring circulating glial fibrillary acidic protein in severe stroke

**DOI:** 10.1136/svn-2025-004645

**Published:** 2025-12-02

**Authors:** Megan Gjordeni, Jakob Pansell, Eric P Thelin, Michael V Mazya, Kaveh Pourhamidi, Perttu J Lindsberg, Olli S Mattila, Charith Cooray

**Affiliations:** 1 Department of Clinical Neuroscience, Karolinska Institute, Stockholm, Sweden; 2 Function Perioperative Medicine and Intensive Care, Karolinska University Hospital, Stockholm, Stockholm County, Sweden; 3 Department of Neurology, Karolinska University Hospital, Stockholm, Stockholm County, Sweden; 4 Department of Clinical Neurophysiology, Karolinska University Hospital, Stockholm, Stockholm County, Sweden; 5 Neurology, University of Helsinki, Helsinki, Uusimaa, Finland; 6 Clinical Neurosciences, University of Helsinki, Helsinki, Uusimaa, Finland; 7 Department of Clinical Neuroscience, Karolinska University Hospital, Stockholm, Stockholm County, Sweden; 8 Department of Clinical Neuroscience, Karolinska Institute, Stockholm, Stockholm County, Sweden

**Keywords:** Stroke, Ischemic Stroke, Cerebral Infarction

## Abstract

Stroke is a global health concern, requiring early and accurate diagnosis for effective treatment. Differentiating between ischaemic stroke and haemorrhagic stroke is critical, as treatment strategies differ significantly. While neuroimaging is the gold standard for differential diagnosis of stroke code patients, blood biomarkers could be a promising and cost-effective diagnostic method for earlier diagnosis in the prehospital setting, where neuroimaging is unavailable. Studies demonstrate that the biomarker glial fibrillary acidic protein (GFAP) can distinguish ischaemic and haemorrhagic stroke with high specificity, though sensitivity varies based on sampling timing and assay methodology. This narrative review explores the potential of GFAP as a diagnostic biomarker in severe strokes and in identifying large vessel occlusions (LVOs). While studies suggest a correlation between higher GFAP levels and stroke severity in haemorrhagic stroke, evidence for this in ischaemic stroke is inconclusive. Combining GFAP with clinical stroke scales and additional biomarkers has shown promise in identifying LVO. Future research should focus on refining the diagnostic role of GFAP in severe strokes, optimising sample timing and including large cohorts representing the full spectrum of stroke severities.

## Introduction

 Stroke is a significant global health concern, ranking as the second-leading cause of death worldwide.^1^ In Sweden alone, 19 923 stroke cases were reported in 2023, of which 16% were deceased at 3 months follow-up.^2^

Stroke has two major subtypes requiring fundamentally different treatment strategies: ischaemic stroke (IS) and haemorrhagic stroke (HS). HS includes intracerebral haemorrhage (ICH) and subarachnoid haemorrhage. Despite their distinct pathophysiological mechanisms, different forms of stroke present with similar clinical manifestations, making early differentiation challenging.^3^ Current guidelines recommend rapid neuroimaging to shorten the time to correct diagnosis and treatment.^4–7^ In the case of IS, rapid intervention with thrombolysis or thrombectomy is essential due to the correlation between functional outcomes and time from symptom onset to thrombolysis and reperfusion.^5 6 8^ ICH patients can benefit from early minimally invasive surgery and medical conservative treatments such as intensive lowering of systolic blood pressure, strict glucose control, antipyretic treatment and rapid reversal of anticoagulation, all associated with improved functional outcomes.^9 10^

Patients with severe stroke are at a substantially higher risk of poor functional outcomes, highlighting the need for rapid diagnosis to guide transport and first prehospital and in-hospital therapeutic interventions.^11^ Higher National Institutes of Health Stroke Scale (NIHSS) scores are strongly associated with large vessel occlusions (LVO); for each 1-point increase in NIHSS score, the risk of having an LVO increases by 15%.^12^ LVOs often carry a poor prognosis with an estimated 90-day mortality rate between 15.3% and 29.2%.^13 14^

Blood biomarkers have been proposed as a cost-effective, time-efficient method to distinguish strokes from stroke mimics (SM) and differentiate IS from HS already in the prehospital setting.^15^ SMs are conditions that present with acute neurological deficits raising the suspicion of stroke but are caused by other conditions such as peripheral vestibular dysfunction or migraines.^16 17^ Glial fibrillary acidic protein (GFAP) is one of these promising protein biomarkers of stroke diagnosis.^18^ GFAP is the main intermediate filament protein of astrocytes within the central nervous system.^19^ In stroke and traumatic brain injury, cell death and neuronal degeneration cause rapid release of GFAP that can be measured in cerebrospinal fluid and blood.^20–22^ Most notably, the release of GFAP is most rapid in HS, which is known to induce early blood-brain barrier (BBB) disruption, while the release is significantly slower in IS, helping to differentiate the two in the early timeframe after symptom onset.^23^

While there are several systematic reviews and meta-analyses on the ability of GFAP to diagnose stroke and discriminate between stroke subtypes,^18 24–29^ the literature is lacking a timely review focusing specifically on liberation of GFAP in patients with severe stroke symptoms. This narrative review aims to:

- Provide an overview of the current evidence on GFAP as a diagnostic blood biomarker in severe stroke.
- Explore the literature analysing the kinetics in blood of GFAP.
- Scrutinise whether GFAP can be used to identify severe stroke with a high likelihood of LVO.

### Search strategy and selection criteria

We performed a narrative review by searching electronic databases, such as PubMed and Google Scholar, to identify relevant articles published up to July 2025. The search used combinations of the keywords “glial fibrillary acidic protein”, “stroke”, “blood biomarker”, and “diagnosis”. Articles were screened for relevance based on title and abstract, followed by full-text review. Studies were eligible for inclusion if they were peer-reviewed, published in English, involved human subjects and reported on GFAP in the context of stroke diagnosis. Studies were excluded if they included non-human subjects, published in other languages than English, or did not provide relevant data on GFAP in stroke diagnosis. Data extracted included study design, patient population, GFAP measurement methods and key findings.

The aim of this narrative review was to investigate GFAP liberation in severe stroke patients. However, during the literature search, no original studies were identified that exclusively examined GFAP in severe stroke cohorts. As a result, all studies on GFAP in stroke, regardless of stroke severity, were included. Data regarding cohort characteristics and stroke severity were evaluated and findings were synthesised with a particular focus on their implications for severe stroke.

### GFAP as a diagnostic blood biomarker

Several studies have confirmed the ability of GFAP to discriminate between ICH and IS with overall high specificity and moderate sensitivity, mainly performed in blood samples collected in the in-hospital setting (table 1).^21 30–34^ Of the six studies referenced here, five used cut-off values ranging between 0.29 and 0.43 ng/mL, while Luger *et al* used 0.03 ng/mL.^32^ However, cut-off values cannot be directly compared between studies and different assays used, as no universally accepted GFAP standard is available, leading to significant differences in the calibration of different assays. The diagnostic capability of GFAP has been reviewed in detail in a meta-analysis previously, with the balance of sensitivity and specificity depending significantly on cut-off selection, onset to sample time and assay performance.^25^ Overall, studies reported a high level of diagnostic accuracy, with sensitivities of 36% to 79% and specificities of 64% to 100%. All cohorts included a majority of IS patients with few to no SM, similar to the six studies included in table 1.^25^

**Table 1:** An overview of six studies aiming to determine GFAP’s discriminatory ability between ICH, IS and SM

		Author					
		Foerch *et al*^30^	Kalra *et al*^31^	Katsanos *et al*^21^	Luger *et al*^32^	Ren *et al*^33^	Rozanski *et al*^34^
Sample (n)	ICH	39	70	34	45	45	25
	IS	163	75	121	146	79	49
	SM	3	10	31	11	n/a	n/a
Total (n)		205	155	186	202	124	74
NIHSS, median (IQR)	ICH	16 (13-20)	19.5 (16-25)	18 (14-22)	12 (7-18)	7 (4-11)	15 (8-18)
	IS	12 (8-18)	12 (5-20)	16 (6-23)	8 (5-16)	4 (1-8)	6 (4-12)
	SM	5	12 (4-21)	n/a	10 (6-12)	n/a	n/a
OST, median (min)	ICH	134	195.0	168	n/a	420	43.0
	IS	123	298.4	179	n/a	600	88.0
	SM	140	162.0	n/a	n/a	n/a	n/a
Cut-off (ng/mL)		0.29	0.33	0.43	0.03	0.34	0.29
Spec. (%)		96.3	90.6	97	94.2	96	100
Sens. (%)		84.2	77.1	91	77.8	61	36.0

For studies including SM, the reported sensitivity and specificity values pertain to the ability of GFAP to differentiate ICH from IS and SM grouped.

GFAP, glial fibrillar acidic protein; ICH, haemorrhagic stroke; IS, ischemic stroke; NIHSS, National Institutes of Health stroke scale; OST, onset-to-sampling time; Sens., sensitivity; SM, stroke mimics; Spec., specificity.

Cohort sizes of the six studies in table 1 are broadly consistent, with five studies including between 124 and 205 participants. This uniformity in size supports comparability across their results. Rozanski *et al* enrolled a smaller cohort (n=74), which may reduce its statistical power, but its inclusion adds important diversity to the evidence base as GFAP measurements were made prehospitally. Overall, the number of included SMs is very few, from 3 to 31, which limits the comparison against IS and ICH.

Another potential use of GFAP as a stroke blood biomarker is to differentiate strokes from SM. As GFAP levels are higher in ICH compared with SM, it can distinguish between these two groups with high accuracy.^21^ One study measured GFAP blood levels in 331 patients covering a broad spectrum of acute and chronic neurological diseases, including typical SM diagnoses. Some of the diagnoses with the highest mean GFAP values were bacterial meningitis and status epilepticus.^35^ High-grade gliomas have also been associated with elevated GFAP.^36^ However, these levels were well below the reported GFAP levels of ICH patients, and these diagnoses do not typically present with stroke-like symptoms. This suggests that most neurological diseases are unlikely to confound the use of GFAP as a biomarker for ICH. Three studies have compared GFAP concentrations between SM and stroke patients (IS and ICH combined), without showing a significant difference.^21 30 32^ This may be because groups of stroke patients are generally comprised primarily of IS patients, who have lower GFAP blood concentrations than ICH.^28 37^ Thus, studies show that average GFAP levels in unselected stroke patients are relatively near to those in mimics.

### Kinetics of GFAP

Normal GFAP blood concentrations are generally low in healthy individuals, with increasing levels with age.^38–40^ Agnello *et al* reported a range between 10.4 and 92.0 pg/mL in their cohort of 334 individuals.^40^ There are several comorbidities which have shown elevated GFAP in blood, which can potentially confound the diagnostic accuracy of GFAP in the clinical setting. Axelsson *et al* compared GFAP blood concentrations in 110 patients with chronic kidney disease stages 3 and 4 with healthy controls and found their levels to be significantly higher.^41^ GFAP levels are also elevated in patients with stable epilepsy, neurodegenerative diseases and traumatic brain injury.^42–44^

A study by Dvorak *et al* aimed to characterise the diagnostic time window of GFAP for differentiating ICH and IS.^23^ Sixty-three stroke patients underwent blood sampling hourly from 1 to 4 hours after stroke onset, as well as 6, 12, 24 and 48 hours thereafter. The optimal time window for differentiating ICH and IS by GFAP was within 2 to 6 hours (diagnostic accuracy 0.83–0.89), due to the higher levels in ICH patients in the acute phase. At the 1-hour sampling time point, the GFAP blood concentration also differed significantly between the IS and ICH group. However, the samples collected at this time were the fewest (14 IS vs 7 ICH), which can explain the lower diagnostic accuracy (0.71). The optimal time window of 2 to 6 hours can be compared with the median onset-to-sampling times (OSTs) in several important studies, presented in table 1.

**Figure 1:**
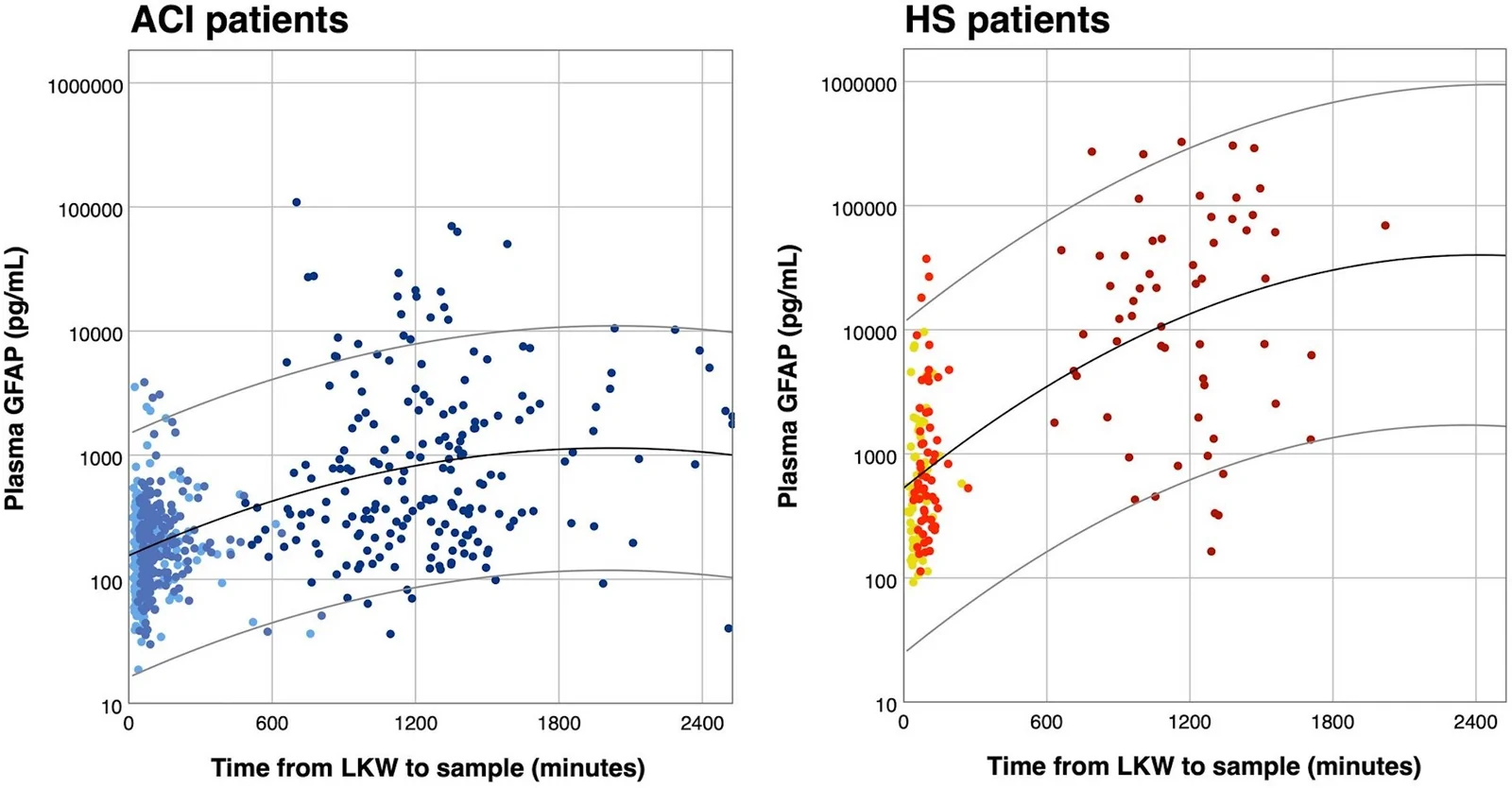
The kinetics of plasma GFAP of IS and HS patients measured at three different time points, prehospitally, at hospital admission and next morning samples, represented by increasing colour saturation. The y-axis is a logarithmic scale of plasma GFAP and the x-axis represents time from ‘last known well’ (LKW) to blood sampling. Figure originally from *Ultra-Early Differential Diagnosis of Acute Cerebral Ischemia and Hemorrhagic Stroke by Measuring the Prehospital Release Rate of GFAP*, by Mattila *et al*^46^ Licensed under Creative Commons BY 4.0.^61^ Modified from original. Cropped to show only the graphs above. ACI, acute cerebral ischaemia; GFAP, glial fibrillar acidic protein; HS, haemorrhagic stroke; IS, ischaemic stroke.

Regarding the kinetics of GFAP over a longer time, it has been shown to peak on the day of symptom onset and subsequently to decrease over the following weeks.^21 45^ Figure 1 shows a representative example of the kinetics of GFAP, including 203 patients with IS and 60 with ICH. Samples were taken at three different time points: at the prehospital stage, at hospital admission and next morning samples.^46^

In summary, the baseline GFAP blood concentration is initially low in healthy, younger subjects. In the instance of HS, it reaches high levels early after symptom onset, while in IS, the release rate is slower, and the attained overall GFAP blood concentration is lower (figure 1).

### GFAP, severe strokes and LVOs

A few studies have investigated whether high NIHSS scores correlate to higher blood concentrations of GFAP. Foerch *et al* have described such an association for ICH patients but not in IS, while Ren *et al* found the opposite.^30 33^ While these two studies have contradicting results, it is important to acknowledge that Ren’s IS group had a median NIHSS of 4 and the ICH group a median of 7; thus, both cohorts include less severe strokes. In the cohort reported by Foerch *et al*, higher median NIHSS of 12 for IS and 16 for ICH were observed. Two other studies, conducted solely on IS patients, described a correlation between NIHSS and blood GFAP concentration.^45 47^ Ferrari *et al* had a cohort of 36 IS patients with a median NIHSS of 6, and Puspitasari *et al* included 64 patients with 59.4% having moderate strokes (NIHSS 4–15).^45 47^ The studies that show a correlation between GFAP blood concentration and NIHSS in IS have generally had a longer OST compared with the studies that did not. As GFAP has a slower release rate in IS compared with ICH, this may affect the results.^23 45^

The studies in table 1 have a range of median NIHSS scores ranging from 4 to 20. However, only a few of the studies have NIHSS score distributions that represent a large span of stroke severity. To accurately compare their results between each other and to be able to draw general conclusions regarding the correlation of NIHSS and GFAP, it would be favourable to have a large cohort including the full continuum from very mild to very severe strokes. Concerning the different temporal profiles of GFAP in ICH and IS, it would be optimal to include several sampling time points from prehospital to days after symptom onset.

ICH volume, a marker of stroke severity, and its association to circulating GFAP has also been studied.^32^ Haematoma volume has been shown to be correlated to NIHSS on admission, as larger bleedings present with initially more severe, but often rapidly worsening symptoms.^46 48^ By using a cut-off value of GFAP 0.29 ng/mL, haemorrhage volumes >15 mL could be detected among an unselected group of stroke patients, including HS and IS, with a sensitivity of 61.5% and specificity of 98.4%.^34^ It can therefore be envisaged that higher NIHSS correlates to higher blood concentrations of GFAP specifically in HS, which has been shown by Foerch *et al* and Mattila *et al* but not in the study by Ren and coworkers, presumably due to milder HS cases.^30 33 46^

Patients with LVOs tend to present with higher NIHSS scores compared with non-LVOs and therefore make up a larger part of severe strokes.^49 50^ The use of blood biomarkers to detect LVOs among severe strokes would be useful to rapidly transport these patients to the nearest thrombectomy centre. Gaude *et al* included 128 stroke patients and aimed to evaluate the diagnostic accuracy of a blood biomarker panel combined with NIHSS-derived stroke severity scales for LVO detection.^50^ They reported an accuracy of 95% by combining the FAST-ED scale with D-dimer and GFAP (sensitivity 91%, specificity 96%). Both biomarkers significantly contributed to LVO detection regardless of the stroke scale they were combined with. These results have been confirmed in a similar study by Durrani *et al.*^51^ Mattila *et al* showed that GFAP alone could differentiate between LVO and HS, in patients with NIHSS >8, with a sensitivity of 92.7% and specificity of 90.3%.^46^ When comparing only LVO and SVO, GFAP could discriminate between these with a diagnostic accuracy of 77%.^52^ The combination of GFAP and D-dimer for LVO detection has been further studied by Gaude *et al* who aimed to assess the performance of a novel point-of-care test and assess its diagnostic performance in a cohort of 210 patients. They showed a strong positive correlation between the point-of-care test and serum GFAP concentrations. By combining the point-of-care test with clinical assessment, LVOs were detected with a sensitivity of 75% and specificity of 92% (PPV 48%, NPV 97%).^53^

### Clinical implications

The use of GFAP in clinical practice could aid decision-making in the prehospital phase where neuroimaging is not immediately available as well as in resource-limited settings. GFAP has shown a high specificity in distinguishing HS from IS.^25 28^ This could be a useful tool to rule in HS among patients with suspected stroke and initially transport them to the closest stroke centre with a later transfer to neurosurgical facilities if needed.^54^ Combined with pre-hospital stroke severity assessment tools, GFAP could also enhance the accuracy of stroke subtyping in the prehospital settings.^55^ However, these combinations can be compared with solely using clinical assessment scales for detecting LVOs. A systematic review by Antipova *et al* found the diagnostically most accurate clinical assessment scales to be FAST-ED ≥4 (PPV 80%, NPV100%), NIHSS ≥10 (PPV 78%, NPV 99%) and RACE ≥5 (PPV 81%, NP 99%).^56^ As the performances are similar, it puts into question whether the addition of point-of-care tests enhances diagnostic accuracy.

As GFAP elevates with age and several morbidities such as epilepsy, chronic kidney disease and neurodegenerative diseases, clinical implication can be affected. The use of GFAP in triaging of these patients will be complicated as current cut-off values are estimated based on studies that usually exclude these patient populations. If GFAP were to be clinically implemented, there would be a need for algorithms factoring in age and comorbidities. Overall, it is important to note that clinical decisions based on GFAP cannot be made based on the current available evidence.

Circulating GFAP concentration could be relevant in patients presenting with severe symptoms indicative of LVO or HS. In three studies of unselected stroke cohorts, GFAP has contributed to the identification of LVOs by excluding HS. HS has generally higher GFAP levels than LVO, and patients with high concentrations could therefore be transported to the nearest stroke centre for initial stabilisation and bundled care.^50 51 57^ Patients with severe symptoms but lower GFAP levels would signal LVO rather than HS and guide transport to the nearest thrombectomy centre, together with prenotification to help the receiving hospital to prepare for IS.^51^ In two studies that combined GFAP with other blood biomarkers and clinical stroke scales, the sensitivity and specificity for identifying LVOs were 71%–82% and 94%–98%, respectively.^50 51^ When establishing cut-off values for differentiating LVO from HS, it is preferable to minimise false negatives, as LVOs are associated with poorer outcomes and require urgent transportation to the correct facility. However, this approach poses a challenge as it likely increases false positives. HS patients may then be misclassified prehospitally and be delayed appropriate care.

The World Stroke Organization recently published a commentary regarding top priorities for improving stroke prehospital care globally, with one of them being developing point-of-care prehospital diagnostic technologies.^58^ Prehospital point-of-care GFAP sampling in intracranial haemorrhage has been studied by Zylyftari *et al.*^59^ In this cohort of 143 patients, GFAP measurements were fully completed in five patients at the prehospital stage by equipping the ambulance with a small centrifuge and a GFAP point-of-care device. The total sample analysis time, including centrifuging, took 25 min. Short sample analysis times are crucial for the implementation of point-of-care blood-based diagnostics. As of now, there have not been studies done on the cost-effectiveness of GFAP testing in stroke diagnostics.

Kalra *et al* have further validated the use of point-of-care devices for rapid diagnosis of ICH.^60^ In their prospective study, they enrolled 353 patients with symptoms of acute stroke admitted within 6 hours of symptom onset. Blood samples were taken prehospitally, and plasma measurements were made on admission using the i-STAT-Alinity device. Median OST was 90 min. Kalra *et al* found age to be a relevant confounder in their cohort as GFAP values in patients with IS and SM increased with age. Patients were therefore categorised into three even age groups, with separate cut-off values. The reported PPVs decreased with increasing age and ranged between 90.0% and 95.5%. By further limiting the analysis to patients with a NIHSS >6 and symptom onset ≤2 hours, higher diagnostic accuracy was achieved. These findings support the prehospital use of GFAP for identifying ICH, allowing for optimised triage and early initiation of anti-hypertensive treatment and anticoagulation reversal.

### Future research

Given that severe strokes have a higher likelihood of LVO, and rapid identification of LVO is critical for triage to thrombectomy centres, future research should specifically evaluate the ability of GFAP to find LVOs within a cohort of severe stroke patients. It would be valuable to compare the diagnostic accuracy of GFAP alone versus its combination with existing stroke scales for LVO detection.

Cohorts could be divided into subgroups based on NIHSS scores, to evaluate the diagnostic accuracy of GFAP in these different groups. If the diagnostic accuracy of GFAP varies between groups, it might be more useful in some instances of decision-making and differentiation than in others.

To this end, the current findings of the relationship between GFAP concentration and stroke severity in IS are inconsistent. However, evaluating the utility of this correlation in a larger cohort with additional, later sampling time points could support clinical guidelines. Measuring GFAP in whole blood in addition to plasma has already been feasible in point-of-care instruments available in ambulances.^58^ Further exploration of the kinetics of GFAP in severe stroke and LVO would allow for finding an optimal time window for measurement. As GFAP has been combined with D-dimer with promising results, it could also be combined with other biomarkers and clinical assessment tools to optimise its diagnostic and prognostic accuracy.

Given the temporal dynamics of GFAP release, future studies should aim to establish time-dependent diagnostic thresholds. This could potentially improve the sensitivity during very early presentations when GFAP levels in ICH may not have fully elevated. As GFAP increases with age in healthy individuals, this should also be considered in future algorithms.

Evaluating the association between GFAP and NIHSS has provided indirect insight into the biomarker’s correlation to tissue damage. This relationship could be further explored by correlating GFAP levels to core infarct volume and penumbra size. Longitudinal studies could evaluate the potential of GFAP as a monitoring biomarker and not only a diagnostic tool. If patterns of GFAP change could predict haemorrhagic transformation in IS, it could potentially have clinical significance.

Before clinical implementation, there are several aspects to be considered. There is a need to develop point-of-care testing methods for rapid diagnosis in prehospital and resource-limited settings. The feasibility and cost-effectiveness of this way of management needs to be evaluated. Currently, studies have predominantly used research assays to analyse GFAP as clinical assays have not been widely introduced. However, blood GFAP assays for rapid clinical use are available in North America and the UK using, for example, the Abbott i-STAT or Alinity, but are awaiting final approval in, for example, the rest of Europe. Likely, several clinical assays will be available to measure blood GFAP levels in the near future.

## Conclusions

Measuring circulating concentrations of GFAP shows promise as a diagnostic tool in stroke, especially for ruling out HS. However, its utility in severe stroke should be further explored. Future research should prioritise: (1) evaluating the accuracy of GFAP in finding LVOs among severe stroke patients; (2) optimising the GFAP measurement time window and evaluating it in NIHSS subgroups; (3) combining GFAP with other biomarkers, perhaps in a panel setting, for enhanced accuracy; and (4) determining feasibility and cost-effectiveness for point-of-care use. This would improve our understanding of the clinical utility of GFAP in severe stroke. As of now, clinical decisions in stroke triaging using GFAP cannot be made based on the available evidence.

## Data Availability

As this is a review synthesising findings from other original studies, there are no data to be shared.
